# Group Cognitive Behavior Therapy Reversed Insula Subregions Functional Connectivity in Asthmatic Patients

**DOI:** 10.3389/fnagi.2017.00105

**Published:** 2017-04-18

**Authors:** Yuqun Zhang, Yuan Yang, Rongrong Bian, Yingying Yin, Zhenghua Hou, Yingying Yue, Huanxin Chen, Yonggui Yuan

**Affiliations:** ^1^Department of Psychosomatics and Psychiatry, ZhongDa Hospital, School of Medicine, Southeast UniversityNanjing, China; ^2^School of Medicine, Institute of Psychosomatics, Southeast UniversityNanjing, China; ^3^Department of Respiration, ZhongDa Hospital, Southeast UniversityNanjing, China; ^4^Key Laboratory of Cognition and Personality, Ministry of Education, School of Psychology, Southwest UniversityChongqing, China

**Keywords:** asthma, cognitive behavior therapy, insula cortex, fMRI, depression

## Abstract

**Background:** Group cognitive behavior therapy (GCBT) is an effective treatment in improving self-management behaviors and quality of life for asthmatic patients. However, the mechanisms by which GCBT improves asthma-related clinical symptoms remain unknown. Previous studies have indicated that insula is an important region involved in the neuropathology of asthma. Therefore, we examined the possible alteration of functional connectivity (FC) in insula subregions after GCBT in asthmatic patients.

**Methods:** Forty-two asthmatic patients and 60 healthy controls (HCs) received resting-state functional magnetic resonance imaging (rs-fMRI) scan and clinical assessments, 17 asthmatic patients completed GCBT treatment consisting of 8 sessions, and then received rs-fMRI scan and clinical assessments.

**Results:** Asthmatic patients had greater left ventral anterior insula (vAI) FC with the left cerebellum posterior lobe, right middle temporal gyrus, and bilateral anterior cingulate cortex (ACC), but less FC with bilateral postcentral gyrus, bilateral occipital lobe, and left precentral gyrus compared with HCs. FC between left posterior insula and left medial frontal gyrus also increased in the patients. In addition, right vAI showed increased FC with right caudate and left putamen. FC between right dorsal anterior insula (dAI) and left calcarine however decreased. The increase in FC in insula subregions were significantly improved following GCBT. FC between the left vAI connectivity and left postcentral gyrus was positively correlated with the percentage of improvement in 17-items Hamilton depression rating scale scores, and FC between the right dAI and left calcarine was negatively associated with the improvement percentage in asthma control test scores.

**Conclusions:** This study in the first time demonstrated that GCBT led to significant improvement of FC between insula subregions and other brain regions.

**Clinical Trial Registration:** An investigation of therapeutic mechanism in asthmatic patients: based on the results of Group Cognitive Behavioral Therapy (Registration number: ChiCTR-COC-15007442) (http://www.chictr.org.cn/usercenter.aspx).

## Introduction

Bronchial asthma is a common multifactorial chronic inflammatory respiratory disease that results in reversible airflow restriction. To et al. (2012) combined data from the World Health Survey collected in 2002–2003 to generate global estimate of asthma burden, which suggested that asthma prevalence ranged from a low of 1.04% in Vietnam to 21.51% in Australia. In China, a recent epidemiological study reported that the prevalence of asthma is 39.52% (Ding et al., 2016). The prevalence of depression has reached 13.41% among Chinese asthmatic patients (Liu et al., 2014). And depression increases the risk of asthma in adults (Tousman et al., 2006). Previous study demonstrated that group cognitive behavioral therapy (GCBT) can improve asthma-related quality of life and depression symptoms (Yorke et al., 2015). However, little is known about the mechanisms.

With the development of neuroimaging techniques, it has become increasingly possible to observe the neurobiological underpinnings of psychotherapeutic interventions. To the best of our knowledge, GCBT-related neuroimaging studies in asthmatic patients have not yet been reported. Our previous study demonstrated that successful GCBT is associated with a reversed effect on the coordination of spontaneous brain activity in the bilateral occipital lobe and sensorimotor cortex (Zhang et al., 2017). Using functional magnetic resonance imaging (fMRI), the relationships between GCBT and functional changes in certain brain regions were also found in patients suffering from depression (Yoshimura et al., 2014) and anxiety disorder (Doehrmann et al., 2013). These studies advanced our understanding of the mechanisms by which GCBT improves symptoms.

FMRI also holds a great promise for finding the mechanisms underlying the diseases and testing new treatments (Pattinson, 2015). Rosenkranz et al. (2005, 2012) explored the relationship between asthma and brain by fMRI and found that insula was hyper-responsive to asthma-related emotional and afferent physiological symptoms. Banzett et al. (2000) also reported that the perception of dyspnea activated the insula cortex in healthy subjects. Insula as a “cortical hub” receives afferent input from lamina I spinothalamocortical tract, carries information pertaining to shortness of breath, and has strong connections with neural structures essential in processing emotional information (Liotti et al., 2001; Rosenkranz et al., 2005; Nieuwenhuys, 2012; Borsook et al., 2016). However, those previous studies were mostly focused on the local function of insula (Banzett et al., 2000; Rosenkranz and Davidson, 2009; Rosenkranz et al., 2012), its connectivity with other brain regions was not addressed. Furthermore, insula is divided into several subregions, including ventral anterior region which is related to chemosensory and socioemotional processing, dorsal anterior region which is involved in high cognitive processing, and posterior region which is associated with pain and sensorimotor processing (Li et al., 2015). The first aim of this study sought to explore if the insula functional connectivity (FC) of asthmatic patients was abnormal. The second aim was to investigate the therapeutic mechanism of GCBT for asthma. We deduced that asthmatic patients would exhibit abnormal insula subregions FC compared with healthy controls (HCs). Certain abnormal FC in asthmatic patients would be possibly reversed or normalized after GCBT.

## Materials and methods

### Participants

After consulting the statistical professor, calculating the sample size, and referencing previous related-studies (Klumpp et al., 2013; Yoshimura et al., 2014; Mason et al., 2016), 42 patients with a diagnosis of bronchial asthma without acute attacks and 60 HCs were recruited. Twenty-one asthmatic patients volunteered to participate in 8 weeks of GCBT, only 17 patients completed the whole course. The average age of 17 patients was 50.76 years (standard deviation = 12.15) with 7 were males and 10 were females.

This study was carried out in accordance with the recommendations of the ethics committee (Zhongda Hospital, Southeast University, Nanjing, People's Republic of China) with written informed consent from all subjects. All subjects gave written informed consent in accordance with the Declaration of Helsinki.

### Inclusion/exclusion criteria

Participants who met the following criteria entered subject group: (1) were all at least 18 years old; (2) were right-handed; (3) had an educational level of junior high school at least; (4) met the diagnostic criteria of bronchial asthma and during non-acute attacks; (5) HCs were required to have a score below 7 on the 17 items of the Hamilton Depression Rating Scale (HDRS-17).

Participants were excluded if they presented with other serious physical diseases, psychotic disorders, alcohol, or drug dependence or are pregnant, lactating. Participants with implanted electronic or other metal devices (such as a cardiac pacemaker, defibrillator, or stent) were also excluded.

### Evaluations

#### HDRS-17

All subjects including both test and control groups were given HDRS-17 evaluation. GCBT group was given another time at the end of 8-weeks GCBT. HDRS-17 (Hamilton, 1960) contains 17 variables which are measured on five-point scales, and it is used to assess the depression severity. Participants with a score equal or above 7 are considered to have depression.

#### Asthma control test (ACT)

All asthmatic patients completed ACT. GCBT group did again at the end of 8-weeks GCBT. ACT (Nathan et al., 2004) contains 5 items with a total score arranging from 0 to 25. Patients with a total score below 20 are thought to have uncontrolled asthma.

### Intervention

GCBT group received GCBT once a week for 1 h over an 8-week period and received GCBT according to the cognitive behavioral therapy manual developed by Beck (2011). GCBT consisted of 8 sessions, with each session lasting approximately 60 min weekly. It was delivered in a small group of 6–8 patients. The content of sessions was focused on helping patients acquire asthma-related knowledge, teaching them methods to identify and modify irrational thoughts that might deduce asthma exacerbation and maintenance, enhancing problem-solving strategies and decision-making skills to deal with practical issues of asthma control, and managing medication use and favorable behaviors. The detailed content of GCBT was displayed in Table 1.

**Table 1:** **Outline of GCBT**.

**Session**	**Main theme**	**Content**
1	Therapeutic alliance	Build therapeutic alliance Introduce GCBT program and therapeutic principles
2	Asthma and psychological	Discuss the relationships between asthma and psychology Discuss asthma-related emotion and its reflex arc
3	Asthma knowledges	Acquire asthma-related knowledge including the concept of asthma, clinical symptoms, mechanisms, treatments and preventions
4	Medicine and self-management	Discuss the rational use of drugs Discuss appropriate self-management behaviors
5	Emotion, thoughts and behaviors	Discuss the role of thoughts on emotion and behaviors Identify functional and dysfunctional thoughts related to asthma exacerbation
6–7	Cognitive rebuilding	Train patients cognitive restructuring skills and techniques for modifying irrational thoughts that may affect their emotions and deduced or deteriorated asthma
8	Problem solving and separation	Teach problem-solving strategies and decision-making skills of asthma control Share future plans Discussing separation and helping patients building confidence

### Brain image acquisition

Imaging was performed on a 3-Tesla Scanner using a homogeneous birdcage head coil. Participants were required to keep their eyes closed, awake, and not think of specific things during scanning. Participants lay supine with the head snugly fixed by a belt and foam pads to minimize head motion. A gradient-recalled echo-planar imaging (GRE-EPI) pulse sequence was set up to acquire resting-state images. For each data volume, we acquired 36 continuous axial-slices in descending order with 3.75 × 3.75 mm in-plane resolution parallel to the anterior commissure-posterior commissure line, 3 mm slice thickness and a 0 mm gap using resting-state imaging (TR = 2,000 ms, TE = 25 ms, flip angle = 90°, acquisition matrix = 64 × 64, field of view = 240 × 240 mm). This acquisition sequence generated 240 volumes in 8 min.

### Functional imaging preprocessing

All image data were reconstructed and inspected by two experienced radiologists. Image preprocessing was performed using the DPARSF software (Chao-Gan and Yu-Feng, 2010). The first 10 time points were discarded for scanner calibration and for subjects to get used to the circumstance. The remaining time points were corrected for timing differences between slices and for motion effects (six-parameter rigid body) using a reference volume in the center of the run. After head motion correction, participants with head motion of more than 2.5 mm of maximum displacement in any direction (x, y, or z) or 2.5° of angular motion were ruled out. The resulting images were spatially normalized into a standard stereotaxic space using a 12-parameter affine approach and an EPI template image that was resampled to 3 × 3 × 3 mm^3^ voxels. Following this, temporal filtering (0.01 Hz < f < 0.08 Hz) was applied to the time series of each voxel to reduce the effect of low-frequency drifts and high-frequency noise. Any linear trend was then eliminated.

### Selection of region of interest (ROI)

The regions of interest (ROIs) were selected according to the previous research (Deen et al., 2011), in which insula was divided into ventral anterior insula (vAI), dorsal anterior insula (dAI), and posterior insula (PI) for both right and left. The bilateral insula subregions were defined anatomically by drawing insula gray matter on the Montreal Neurological Institute (MNI) 152 standard brain. Each voxel in the insula subregion ROIs (converted to 3-mm resolution) was used as a seed in a whole-brain functional connectivity analysis in both asthmatic patients and HCs.

### FC analysis

The FC analysis was supported by REST tool kit (http://www.resting-fmri.sourceforge.net) (Song et al., 2011). Global trend, white matter (WM) and cerebrospinal fluid (CSF) were obtained by averaging the time series within the whole brain, WM and CSF masks, respectively. For each insula-ROI, a seed referenced time course was obtained by averaging the time series of all voxels in the ROI. Then Pearson's correlation analysis was performed between the seed reference time course and time series of each voxel in the brain in a voxel wise way. And a Fisher's z-transform was applied to improve the normality of the correlation coefficients (Lowe et al., 1998). Six head motion parameters and the mean time series of global signals, WM signals and CSF signals were introduced as covariates into a random effects model to remove possible effects of head motion, global signal, WM signal and CSF signals on the results.

### Statistical analysis

Predictive Analytic Software (PASW) Statistics 18 package was employed (IBM Corporation, Armonk, NY, USA) to complete the analyses. Age, education, and HDRS-17 were analyzed by independent-samples *t*-test. Gender was compared by means of the Chi-square test. Paired-samples *t*-test was used to compared the scale scores before and after GCBT. *P* < 0.05 were considered to indicate statistical significance.

To determine the underpinnings of GCBT in the context of asthma, an independent-samples *t*-test was performed on the groups' insula subregions FC maps in a voxel-by-voxel manner, with age, gender, and education level as covariates at baseline. AlphaSim correction based on Monte Carlo simulation algorithm was used to correct for multiple comparisons [single voxel *P* = 0.005/6, FWHM = 6 mm, with 61 × 73 × 61 mm^3^ gray matter mask, which yielded a corrected threshold of *P* < 0.005, cluster sizes > 945 mm^3^, (http://afni.nimh.nih.gov/pub/dist/doc/manual/AlphaSim.pdf)]. Then, abnormal brain regions by baseline comparison with controls were selected as seeds for a subsequent paired-samples *t*-test between pre- and post-GCBT scores. AlphaSim correction was also used to correct for multiple comparisons (single voxel *P* = 0.005, FWHM = 6 mm, with selected masks, which yielded a corrected threshold of *P* < 0.005, cluster sizes > 189, 81, 108, 108 mm^3^ for the FC of left vAI, left PI, right vAI, and right dAI respectively). The analyses were performed using the REST extract ROI series also from REST tool kit. Pearson's correlation analyses were then performed to examine correlations between abnormal brain regions' *z*-values and psychological performance of asthmatic patients using PASW 18.0 software.

## Results

### Demographic and clinical data

Table 2 showed the detailed demographic information and scale scores before and after GCBT. Patients were significantly older than HCs (*P* < 0.05) which would be a covariate in the following statistical analysis of insula FC. There were no significant differences between asthmatic patients and HCs in terms of gender and education level. HDRS-17 scores of patients were significantly higher than HCs' (*P* < 0.001). After GCBT, patients showed significantly higher ACT scores and lower HDRS-17 scores (*P* < 0.001).

**Table 2:** **Demographics and clinical characteristics of asthmatic patients and health controls**.

	**Asthmatic patients (*N* = 42)**	**Health control (*N* = 60)**	***P-*****value**
Age (years)	51.88 ± 9.96	45.78 ± 14.49	0.020^a^
Gender (male/female)	18/24	24/36	0.773^b^
Education (years)	11.81 ± 2.58	12.42 ± 3.57	0.384^a^
Duration (years)	22.03 ± 19.44	–	
HDRS-17 scores	6.00 ± 5.37	0.93 ± 1.34	<0.001^a^
ACT scores	17.62 ± 4.86	–	
	**Before GCBT (*N* = 17)**	**After GCBT (*N* = 17)**	***P-*****value**
ACT scores	16.47 ± 4.27	21.05 ± 4.28	0.001^c^
HDRS-17 scores	5.59 ± 5.40	1.82 ± 2.35	<0.001^c^

*Data are expressed as mean ± standard deviation*.

a *independent-samples t-test*;

b *Chi-square test*;

c *paired-samples t-test*.

*HDRS, 17-items Hamilton Depression Rating Scale; GCBT, group cognitive behavior therapy; ACT, asthma control test*.

### FC results in asthmatic patients and HCs at baseline

**Figure 1:**
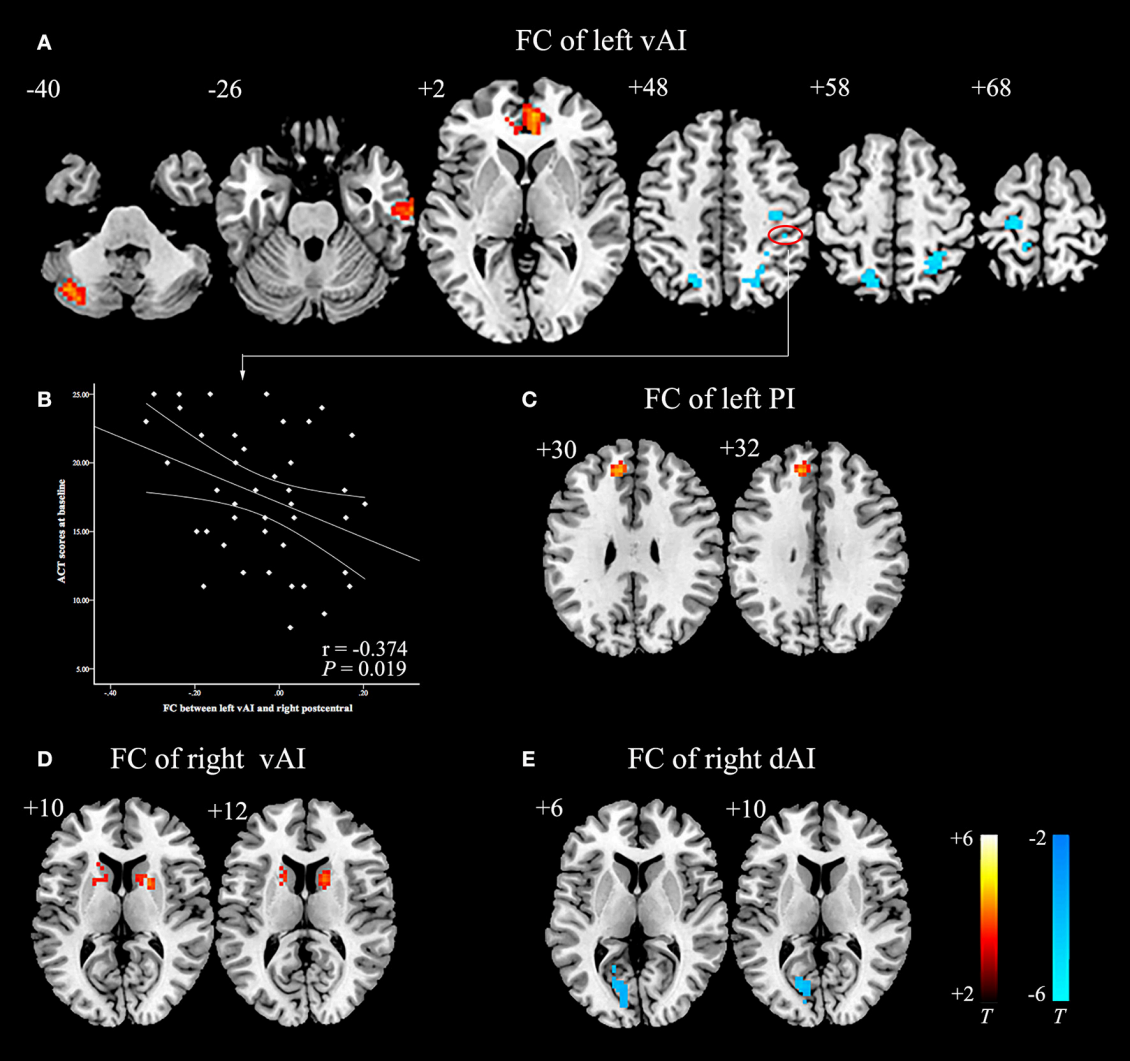
**Abnormal insula subregions FC in asthmatic patients compared with HCs at baseline. (A)** The asthmatic patients showed significant increased FC between the left vAI and left cerebellum posterior lobe, right middle temporal gyrus, and bilateral anterior cingulate cortex respectively. And they also showed significant decreased FC between left vAI and bilateral postcentral gyrus, bilateral occipital lobe and left precentral gyrus (*P* < 0.005/6, AlphaSim corrected). **(B)** Decreased FC between left vAI and right postcentral gyrus was negatively correlated with ACT scores in asthmatic patients. **(C)** The asthmatic patients showed significant increased FC between the left PI and left medial frontal gyrus (*P* < 0.005/6, AlphaSim corrected). **(D)** The asthmatic patients displayed significant increased FC between right vAI and right caudate, left putamen (*P* < 0.005/6, AlphaSim corrected). **(E)** Significant decreased FC between right dAI and left calcarine was found in asthmatic patients (*P* < 0.005/6, AlphaSim corrected). The color bars indicated the *t*-value from independent-samples *t*-test between asthma and HCs groups. Abbreviations: FC, functional connectivity; HCs, healthy controls; vAI, ventral anterior insula; PI, posterior insula; dAI, dorsal anterior insula.

Compared with HCs, asthmatic patients showed that the left vAI has an increased FC with left cerebellum posterior lobe, right middle temporal gyrus, and bilateral anterior cingulate cortex (ACC), and decreased FC with bilateral postcentral gyrus, bilateral occipital lobe, and left precentral gyrus (Table 3, Figure 1A). They also showed an increased FC between left PI and left medial frontal gyrus (Table 3, Figure 1C). In the right insula, increased FC was found between right vAI with right caudate and left putamen (Table 3, Figure 1D). A decreased FC was found between right dAI and left calcarine (Table 3, Figure 1E). However, no significant differences of FC in both the left dAI and right PI were found between asthmatic patients and HCs.

**Table 3:** **Brain regions showed altered FC in asthmatic patients compared with HCs**.

**Peak area**	**BA**	**Side**	**MNI coordinates**			**voxels Number**	**Peak *t*-value**
			***X***	***Y***	***Z***		
**FC OF LEFT vAI**							
Cerebellum posterior lobe	−	L	−39	−69	−39	41	4.387
Middle temporal gyrus	21	R	66	−18	−24	50	4.6121
Anterior cingulate cortex	32	B	6	36	6	122	4.5451
Postcentral	6	R	54	−18	21	35	−4.4352
Postcentral	2	R	42	−33	42	71	−4.6205
Parietal Lobe	7	L	−18	−63	54	61	−4.6808
Parietal Lobe	7	R	30	−48	57	111	−4.5955
Precentral Gyrus	4	L	−18	−24	69	38	−4.7301
**FC OF LEFT PI**							
Medial frontal gyrus	9	L	−12	42	33	38	4.5504
**FC OF RIGHT vAI**							
Caudate	−	R	21	6	9	62	4.2601
Putamen	−	L	−24	3	3	40	4.1338
**FC OF RIGHT dAI**							
Calcarine	30	L	−15	−72	9	64	−4.5115

*Threshold was set at P < 0.005/6 (AlphaSim-corrected, cluster size > 945 mm^3^ for left vAI, left PI, right vAI, right dAI respectively). X, Y, Z, coordinates of primary peak locations in the MNI space*.

*MNI, Montreal Neurological Institute space; FC, functional connectivity; HCs, healthy controls; BA, Brodmann area; R, right; L, left; B, bilateral; vAI, ventral anterior insula; PI, posterior insula; dAI, dorsal anterior insula*.

### FC results in asthmatic patients at post-GCBT

**Figure 2:**
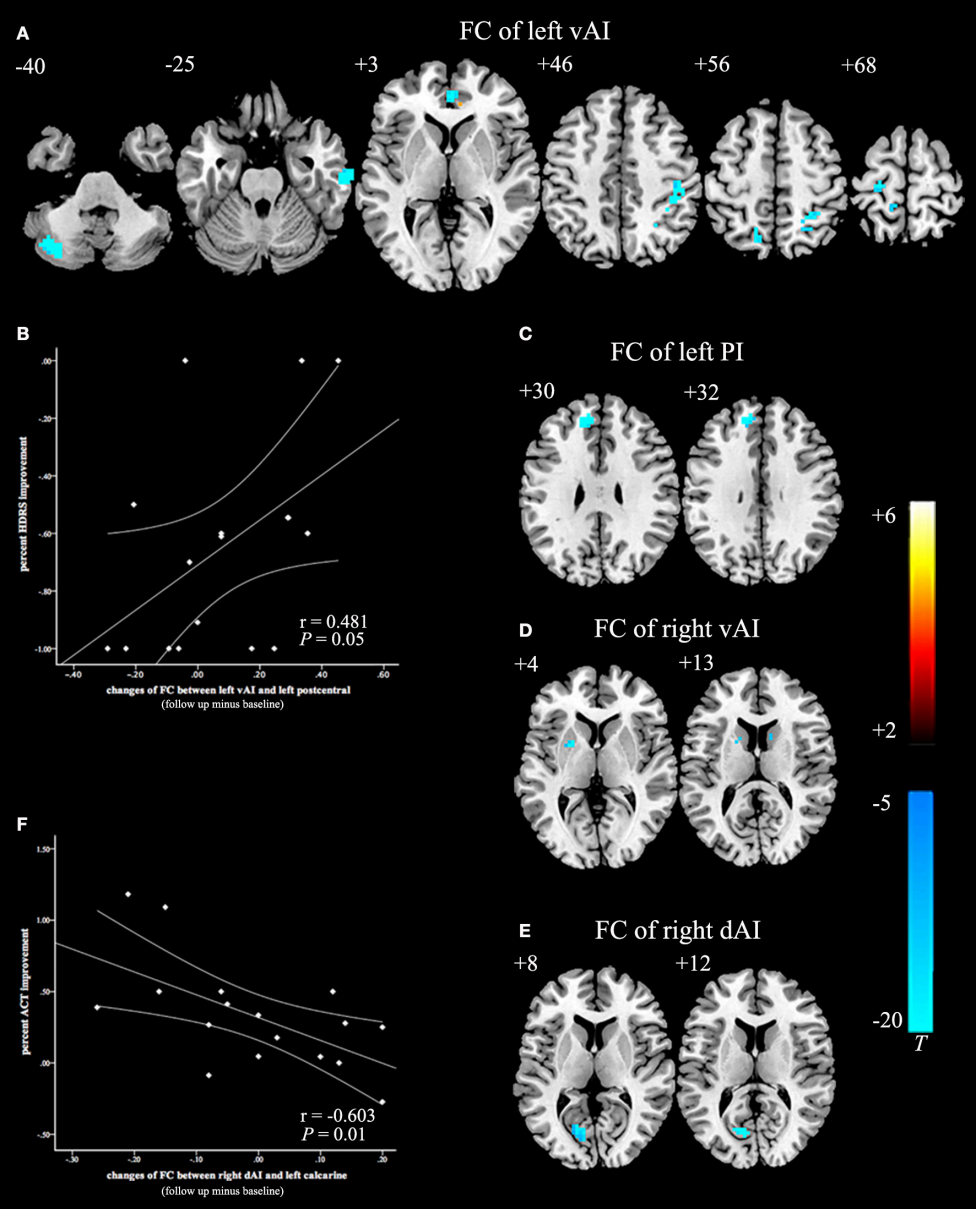
**Paired t-statistic maps of insula subregions FC between post- and pre-GCBT in asthmatic patients (*P* < 0.005, AlphaSim corrected)**. **(A)** Reversed changes displayed in the FC between left vAI and left cerebellum posterior lobe, left temporal lobe, right ACC respectively. And the FC between left vAI with bilateral postcentral gyrus and bilateral occipital lobe were still decreased in asthmatic patients. **(B)** Decreased in left vAI connectivity with left postcentral gyrus following GCBT positively correlated with percent improvement in depression severity (HDRS-17). **(C)** FC between the left PI and left medial frontal gyrus were reversed after GCBT. **(D)** Reversed changes exhibited in the FC between right vAI and left caudate, right putamen respectively. **(E)** After GCBT, asthmatic patients showed decreased FC between right vAI and left calcarine. **(F)** Decreased in right dAI connectivity with left calcarine following GCBT negatively correlated with percent improvement in asthma control (ACT). The color bars indicated the t value from paired-samples *t*-test. Abbreviations: FC, functional connectivity; HCs, healthy controls; vAI, ventral anterior insula; PI, posterior insula; dAI, dorsal anterior insula; GCBT, group cognitive behavior therapy; HDRS-17, 17-itmes Hamilton Depression Rating Scale; ACT, asthma control test.

After 8 weeks of GCBT treatment (Figure 2), FC of left insula in patients was reversed between left vAI and left cerebellum posterior lobe, left temporal lobe, and right ACC (Table 4, Figure 2A). FC between left PI and left medial frontal gyrus were also reversed to the level of pre-GCBT (Table 4, Figure 2C). And FC was reversed between right vAI with left caudate and right putamen (Table 4, Figure 2D). After GCBT, the insula FC with other brain regions (including bilateral parietal lobe, bilateral postcentral gyrus, right precuneus, left precentral gyrus, and left calcarine) were significantly decreased in asthmatic patients (Table 4, Figures 2A,F).

**Table 4:** **Brain regions showed altered FC in asthmatic patients before and after GCBT**.

**Peak area**	**BA**	**Side**	**MNI coordinates**			**voxels Number**	**Peak *t*-value**
			***X***	***Y***	***Z***		
**FC OF LEFT vAI**							
Cerebellum Posterior Lobe	−	L	−33	−75	−39	41	−15.5062
Temporal lobe	20	R	63	−18	−24	22	−11.5868
Anterior cingulate cortex	32	R	3	45	3	17	−7.6196
Anterior cingulate cortex	32	R	9	33	0	8	4.3224
Parietal lobe	43	R	54	−18	21	10	−9.0851
Parietal lobe	7	L	−15	−66	57	17	−5.354
Postcentral gyrus	2	R	39	−36	42	49	−19.721
Postcentral gyrus	3	L	−12	−39	72	12	−6.7005
Precuneus	7	R	36	−45	51	68	−12.4624
Precentral gyrus	4	L	−21	−24	72	11	−8.0639
**FC OF LEFT PI**							
Medial frontal gyrus	9	L	−9	45	30	36	−9.9896
**FC OF RIGHT vAI**							
Caudate	−	R	12	9	15	4	−5.1337
Putamen	−	L	−24	3	3	23	−7.1454
**FC OF RIGHT dAI**							
Calcarine	30	L	−18	−57	6	4	−5.1478
Calcarine	30	L	−9	−72	15	26	−7.9788

*Threshold was set at P < 0.005 (AlphaSim-corrected, cluster size > 189/81/108/108 mm^3^ for left vAI, left PI, right vAI, right dAI respectively). X, Y, Z, coordinates of primary peak locations in the MNI space*.

*GCBT, group cognitive behavior therapy; MNI, Montreal Neurological Institute space; FC, functional BA, Brodmann area; R, right; L, left; B, bilateral; vAI, ventral anterior insula; dAI, dorsal anterior insula; PI, posterior insula*.

### Correlations between FC with depression severity and asthma control

Before GCBT, the FC between left vAI and right postcentral was found negatively correlated with the ACT scores (Figure 1B). After GCBT, the change of FC between left vAI and left postcentral cortex was found positively correlated with the percentage improvement in HDRS-17 scores (Figure 2B). The change of FC between right dAI and left calcarine, however, showed a negatively correlation with the percentage of improvement in ACT scores (Figure 2F).

## Discussions

The current study at the first time reported that GCBT might modify FC between insula subregions and other certain brain regions.

In the present study, compared with HCs, asthmatic patients showed hyper-connectivity between left vAI with left cerebellum posterior lobe, right middle temporal gyrus, and bilateral ACC, and between left PI with left medial frontal gyrus, and between right vAI with right caudate and left putamen. The findings are similar to that reported by Dodd et al. (2012) in patients with chronic obstructive pulmonary disease (COPD) showing, a higher activation in almost all gray matter resting-state networks (including ventral- dorsal network, default mode network, bilateral fronto-parietal network, sensorimotor network, and prefrontal network). In our study, FC between visual network with both left vAI and right dAI were decreased. Taken together, these findings further suggested that asthmatic patients may indeed have widespread neuronal abnormalities. Asthmatic patients showed reduced FC between left vAI and sensorimotor areas, consistent with previous reports in COPD patients showing reduced cortical thickness, surface area (Chen et al., 2016), and deoxyhaemoglobin (Higashimoto et al., 2015) in sensorimotor areas. These studies suggested that abnormal breathing which results in the unbalance of carbon dioxide in cerebrovascular system might influence the function of sensorimotor areas. Interestingly, decreased FC between left vAI and right postcentral gyrus was found to be negatively correlated with asthma control level, which supported that reduced left vAI FC with sensorimotor areas might involve in the impaired breathing pattern.

Straube et al. (2006) found a significant reduction of hyperactivity in the insula and ACC in spider phobic patients after CBT. CBT also decreases the metabolism in the left cerebellum and ACC in the panic patients (Sakai et al., 2006). Neuroimaging revealed that vAI is connected to visceromotor regions that regulates allostasis, as well as regions representing interoceptive and other sensory inputs associated to affective experience (Deen et al., 2011; Touroutoglou et al., 2016). The cerebellum posterior lobe (Paradiso et al., 2003; Wildgruber et al., 2005), temporal lobe (Cauda et al., 2011), and ACC (Weidt et al., 2016) are believed to play essential roles in the integration of multimodal information vital in sensorimotor and emotional. Thus, we proposed that the reversed FC between left vAI and those above brain regions after GCBT might be related to the neurobiological mechanisms underlying improved asthma-related emotion or perception.

Reversed FC between right vAI with right caudate and left putamen after GCBT was consistent with the findings by Baxter et al. (1992) and Schwartz et al. (1996), that patients with obsessive-compulsive disorder showed decreased activation in the caudate after CBT. They demonstrated the idea that the pathological activity of cortical-striate-thalamic circuit can be responsible for the brain mechanism of both fixed, repetitive thoughts and behaviors in obsessive-compulsive disorder (Porto et al., 2009). Caudate is believed to play a crucial role in cognitive (Jaspers et al., 2017), thus, the reversed FC between right vAI and right caudate following GCBT would be possibly associated with the asthma-related twisted thoughts (e.g., catastrophic thoughts, irrational thoughts). In addition, patients with psychosis displayed reduced activation in the left putamen when they faced fearful and angry expression (Kumari et al., 2011). Since vAI is involved in emotional process (Li et al., 2015), the reversed FC between right vAI and left putamen might be associated with the improvement of emotion after GCBT.

Kucyi et al. (2016) explored the neurobiological mechanisms of CBT for healthy subjects who received painful thermal stimuli, suggesting that subjects received CBT shows reversed FC between areas of default mode (DMN) and executive control network (e.g., PI). Morris et al. (2015) detected the changes of brain function with virtual reality exposure therapy-a treatment similar to CBT, founding that the neurophysiological changes in middle frontal gyrus are related to pain catastrophization in patients with fibromyalgia. Since the neural structures that promote dyspnea and pain are shared (Nishino, 2011), thus, GCBT might possibly modulate the asthma through affecting FC between left PI and left medial frontal gyrus.

Also of clinical importance was the GCBT-specific decrease in the left vAI connectivity with left postcentral gyrus, which was correlated with the improvement in depression severity. It suggested that patients exhibiting a strong decrease in FC between left vAI and left postcentral gyrus tended to have a strong improvement of the depression severity with GCBT. This may be attributable to the roles of the left vAI and postcentral gyrus in depression, which show decreased global FC density in patients with depression (Guo et al., 2015; Zhuo et al., 2016). Asthmatic patients also exhibited correlation between decreased FC (connectivity between right dAI and left calcarine) and improvement in asthma control level with GCBT. Madjar et al. (2012) have reported that spontaneous respiration influences the function of the occipital lobe, a region that is characterized by high vascular density. Therefore, it was possible that favorable asthma control could improve abnormal ventilation through changing the function of occipital lobe.

A limitation of the current study was that the groups were not randomized, so we could not rule out effects of selection bias for take-up of GCBT. And this study used a relatively small sample, thus, the insula subregions FC in asthmatic patients with and without depression could not be further compared. In addition, the generalizability of the results might have been reduced due to the sampling strategy. To overcome these limitations, studies with larger sample sizes are needed in the future.

In summary, this study demonstrated that successful GCBT leads to the reorganization of insula subregions FC with certain brain regions. These changes included reversions of pathophysiology detected at baseline and some non-reversed changes. GCBT improved depression severity of asthmatic patients through modulating FC between left vAI and left postcentral, and asthma control level by affecting FC between right dAI and left calcarine.

## Author contributions

YYua was responsibility for experimental design. YZ, YYa, and RB collected the participants. YZ analyzed the data and wrote the manuscript. ZH, YYi, YYue, HC provided language help for the article. All authors reviewed and approved for publication.

## Funding

This work was supported by National Natural Science Foundation of China (grant number 81371488, YYua) and the Fundamental Research Funds for the Central Universities (Southeast University, grant number 2242016K41053).

### Conflict of interest statement

The authors declare that the research was conducted in the absence of any commercial or financial relationships that could be construed as a potential conflict of interest.
